# Editorial for the Special Issue “Microorganisms in Agriculture”

**DOI:** 10.3390/microorganisms14081652

**Published:** 2026-07-29

**Authors:** Wen-Ching Chen

**Affiliations:** International Bachelor Program in Agribusiness, College of Agriculture and Natural Resources, National Chung Hsing University, Taichung 40227, Taiwan; julychen@nchu.edu.tw

The integration of multi-omics approaches—including high-throughput sequencing, whole-genome analysis, and functional genomics—alongside continued advances in biotechnology has enabled agricultural researchers to systematically dissect, at the molecular level, how microorganisms drive elemental cycling, regulate plant growth, suppress diseases, and degrade contaminants. Against this backdrop, the multiple pressures facing global agriculture—including soil degradation, the compounding accumulation of heavy metals and microplastics, and the impacts of extreme climate events on rhizosphere ecology—have further intensified the urgency of developing and deploying functional microorganisms. This Special Issue compiles eleven papers spanning the core themes of plant growth promotion, biological control, bioremediation, and stress resilience, collectively mapping the current research frontiers in agricultural microbiology.

In the area of plant growth-promoting microorganism (PGPM) discovery and evaluation, this Special Issue presents diverse evidence across crop systems and soil environments. Sun et al. [1] reported the first complete genome of *Glutamicibacter endophyticus*, revealing that the strain responds to short-term salt shock via Na^+^/H^+^ antiporters and potassium uptake proteins, while accumulating compatible solutes for long-term osmotic adaptation; the genome also harbors multiple plant growth-promoting (PGP) functional genes. Under salt stress, inoculation increased wheat germination rates from 37.5% to 95% and enhanced maize seedling dry weight by 41.92%, providing a molecular foundation for developing inoculants tailored to saline–alkali soils. Jesus et al. [2] compared the effects of compost extracts with nitrogen-fixing bacteria (*Bradyrhizobium japonicum* + *Azospirillum brasilense*) on soybean, finding that the bacterial inoculant conferred greater photosynthetic efficiency, while compost extracts performed better in 1000-grain weight, soil microbial biomass carbon enhancement, and pest suppression; notably, each treatment outperformed their combination. Stanojković-Sebić et al. [3] used arugula as a model crop and demonstrated that integrated organic fertilization with bioinoculants yielded the highest soil microbial activity, whereas the integration of inorganic fertilizers with bioinoculants increased total yield by 121% over the control, supporting integrated fertilization as the optimal strategy for arugula cultivation.

Four papers on biological control address complementary knowledge gaps regarding multi-functional strains and broad-spectrum biocontrol potential. Moreira et al. [4] showed the multiplex biocontrol potential of *Purpureocillium lilacinum* SBF054: the strain colonized the internal tissues of common bean roots, stems, and leaves, and partially colonized soybean and sunflower roots; in vitro assays showed 86% inhibition of *Rhizoctonia solani* mycelial growth; and the strain demonstrated high virulence against eggs, nymphs, and adults of the stink bug *Euschistus heros*, with cumulative mortality reaching 65–80%, providing experimental support for a single-strain integrated plant protection strategy. Espinosa Bernal et al. [5] isolated *Bacillus altitudinis* CH05 and *Bacillus tropicus* CH13 from pepper seed surfaces and showed that their volatile organic compounds (VOCs) inhibited mycelial growth of *Sclerotinia* sp., *R. solani*, and *S. rolfsii* by 79–91%; GC-MS analysis confirmed 2,5-dimethylpyrazine and acetoin as the major shared active components, providing mechanistic evidence for exploiting seed microbiomes as biocontrol resources. Xu et al. [6] isolated *Streptomyces luteireticuli* ASG80 from sisal roots and confirmed its broad-spectrum inhibitory activity against multiple *Phytophthora* species in vitro; pot experiments demonstrated that its extract controlled sisal zebra disease (*Phytophthora nicotianae*) with efficacy comparable to the fungicide metalaxyl, offering a genome-supported candidate strain for non-chemical management of *Phytophthora* diseases. Nekoval et al. [7] evaluated four antagonistic fungi against the root-knot nematode *Meloidogyne hapla* on tomato; *Paecilomyces lilacinus* and *Arthrobotrys conoides* achieved gall inhibition rates of 91.8% and 88.4%, respectively—comparable to the chemical nematicide Vydate 5G—while simultaneously improving tomato yield and fruit quality, providing direct experimental support for replacing chemical nematicides with biological alternatives.

The response of soil microbiota to emerging contaminants constitutes a third major axis of this Special Issue. Cao et al. [8] developed a three-layer composite immobilization particle using rice husk biochar as the core carrier, combined with calcium alginate and waterborne polyurethane (WPU), to encapsulate *Bacillus aquimaris* within a bilayer protective structure that effectively shields it from competition by indigenous microorganisms; this system reduced the half-life of di-n-butyl phthalate (DBP) degradation to 1.61 days, offering a scalable bioremediation strategy for phthalate ester-contaminated environments. Cruz et al. [9] systematically evaluated the dose–response effects of polyethylene microplastics derived from commercial resins over a 70-day incubation experiment, revealing that high-concentration treatments (7–14%) significantly altered soil pH, phosphorus availability, and bacterial community composition, with three soil enzymes (FDA, ACP, NAG) showing dose-dependent but directionally divergent responses, providing baseline data for ecological risk assessment of microplastic accumulation in farmland. Wang et al. [10] investigated the rhizosphere bacterial community of the Cd hyperaccumulator *Sedum alfredii* under combined polystyrene nanoplastic (PS-NP) and cadmium (Cd) contamination, demonstrating that combined exposure significantly reduced the relative abundance of Actinobacteriota (−19.76%) and increased that of Acidobacteriota (+16.05%) compared to single Cd treatment; functional analysis further indicated suppression of glycan biosynthesis and metabolism, providing rhizosphere ecological evidence for evaluating phytoremediation strategies under co-contamination scenarios.

Taken together, the review by Nie et al. [11] provides the theoretical backbone of this Special Issue: arbuscular mycorrhizal fungi (AMF) enhance nitrogen and phosphorus uptake through their extensive mycelial networks, simultaneously modulating antioxidant enzyme systems and promoting the accumulation of osmoregulatory compounds, thereby conferring multi-layered systemic protection against drought, salinity, heavy metals, temperature extremes, waterlogging, and biotic stress—serving as the overarching integrative framework for the localized effects revealed by the individual studies in this collection. The papers in this Special Issue collectively point toward three priority directions for future research: First, transitioning from single-strain evaluation to synthetic community design, exploring the cooperative stability of multiple functional microorganisms under field conditions; second, establishing threshold models for the loss of microbial function under combined contamination scenarios to support regulatory decision-making in soil ecological risk assessment; and third, re-evaluating the niche adaptability of existing functional strains under climate change scenarios, particularly the mechanisms by which elevated temperature and intermittent drought disrupt plant growth-promoting efficacy. Together, these papers confirm that agricultural microbiology is advancing from descriptive taxonomy toward mechanism-driven precision application, and that the integration of interdisciplinary approaches—from genomics to field agronomy—will be an indispensable pathway toward realizing this transition.
